# 1170. Follow-up of vaccine preventable disease hospitalisations in the ageing population: Loss of Independence

**DOI:** 10.1093/ofid/ofad500.1010

**Published:** 2023-11-27

**Authors:** Ahmed Salem, Maximilian Hartmann, Nathalie Servotte, Emmanuel Aris, T Mark Doherty, Ekkehard Beck

**Affiliations:** GSK, Wavre, Brabant Wallon, Belgium; Institute for Medical Information Processing, Biometry and Epidemiology, Munich, Bayern, Germany; GSK, Wavre, Brabant Wallon, Belgium; GSK, Wavre, Brabant Wallon, Belgium; GSK, Wavre, Brabant Wallon, Belgium; GSK, Wavre, Brabant Wallon, Belgium

## Abstract

**Background:**

Influenza, pneumococcal infection, herpes zoster and pertussis are vaccine preventable diseases (VPDs) that cause significant burden for older adults (50+ years old). Downstream effects beyond the acute phase such as loss of independence have not been well characterised.

**Methods:**

Using Optum’s de-identified Clinformatics® Data Mart Database, a retrospective claims database study was conducted to assess the impact of a VPD hospitalisation on subsequent loss of independence over 365 days of follow-up during the epidemiological years 2016–2018. Loss of independence is a composite endpoint defined as either a change in residence status from “living at home” to “long-term care facility” or the need of home health/home care (HHHC) which was not needed at baseline. Both were assessed over 365 days of follow-up after a VPD-hospitalisation. The VPD-hospitalised cohort were subjects hospitalised due to VPD as primary or secondary diagnosis or both. Controls were matched at baseline on variables like demographics, insurance, comorbidities, and Charlson Comorbidity Index (CCI) score. Results were stratified by age and CCI category at baseline and reported as mean difference between cohorts (with 95% confidence interval, CI).

**Results:**

Subjects hospitalised for a VPD would experience at least 20% (18.9–21.5%, p< 0.001) more LoI versus their matched counterparts over 365 days (Figure 1). Furthermore, at least 12% (10.0–13.2%, p< 0.001) more subjects in the VPD-hospitalised cohort experienced a worsening in their residence status (Figure 2) at 365 days of follow-up. Finally, the need for HHHC was more pronounced for subjects hospitalised for a VPD where a mean difference of at least 9% (8.1–9.3%, p< 0.001) and a maximum of 27% (25.0–28.7%, p< 0.001) was observed compared to controls (Figure 3).

**Figure 1:**
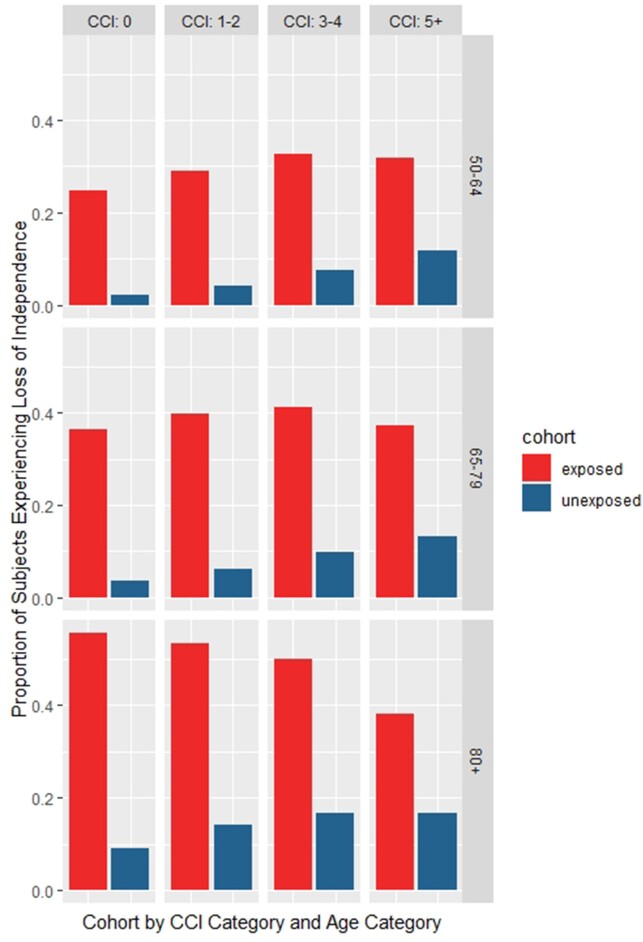
Loss of inpendance

**Figure 2:**
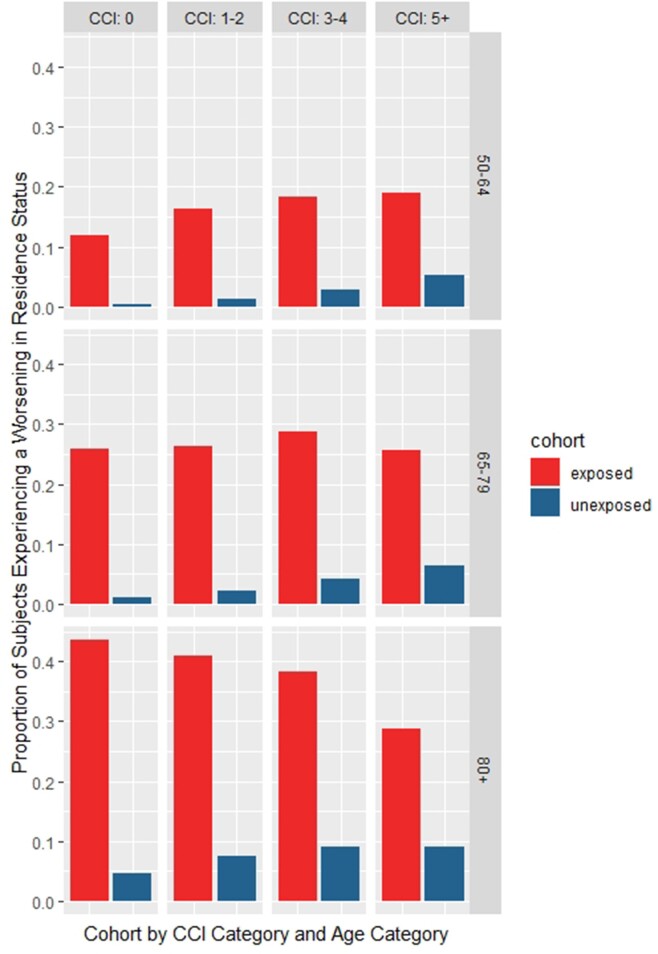
Proportion with worsening in residence status

**Figure 3:**
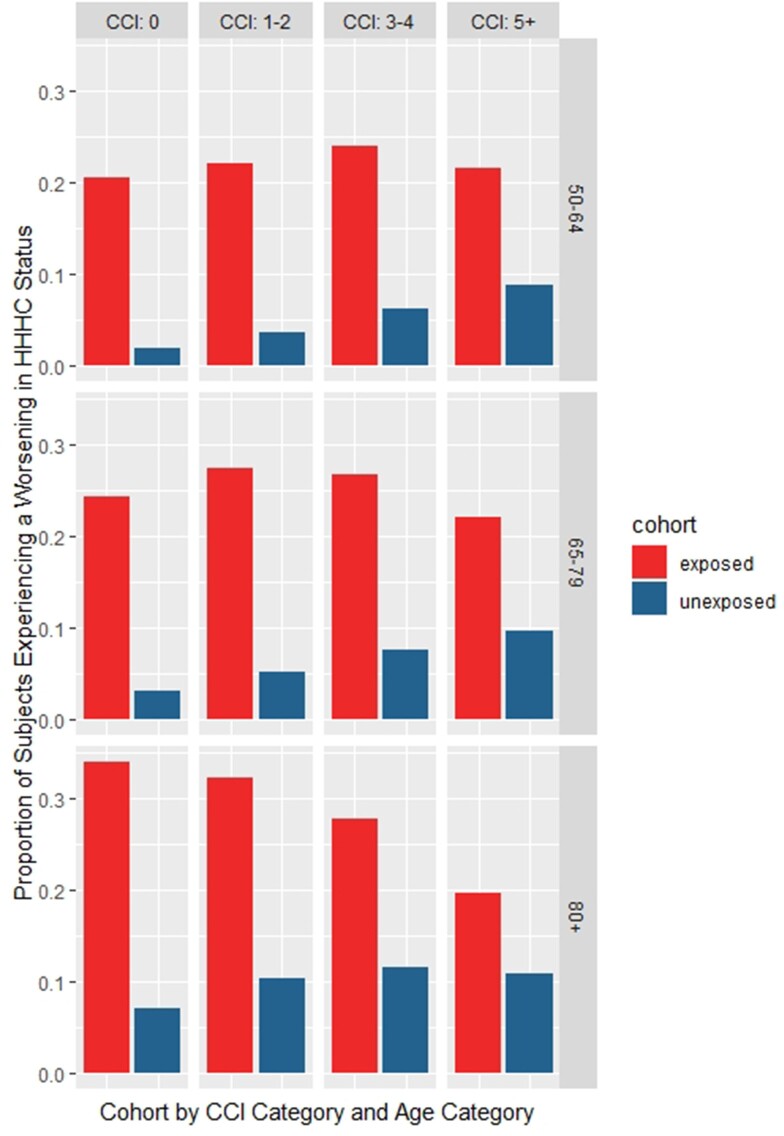
Experiencing worsening in HHHC Status

**Conclusion:**

Individuals hospitalized for a VPD were more likely to experience a loss of independence as defined by change in residence status and the need for home health care compared to matched controls. These findings highlight the potential burden of VPDs beyond the acute disease phase and can provide important motivation to improving preventive programs including vaccination.

**Disclosures:**

**Ahmed Salem, MSc**, GSK: employee|GSK: Stocks/Bonds **Maximilian Hartmann, PhD**, GSK: Grant/Research Support **Nathalie Servotte, PhD**, GSK: employee|GSK: Stocks/Bonds **Emmanuel Aris, PhD**, GSK: employee|GSK: Stocks/Bonds **T. Mark Doherty, PhD**, GSK: employee|GSK: Stocks/Bonds **Ekkehard Beck, PhD**, GSK: employee|GSK: Stocks/Bonds

